# *N*-Dealkylation of Amines

**DOI:** 10.3390/molecules27103293

**Published:** 2022-05-20

**Authors:** Ali Alipour Najmi, Rainer Bischoff, Hjalmar P. Permentier

**Affiliations:** Department of Analytical Biochemistry, Groningen Research Institute of Pharmacy, University of Groningen, A Deusinglaan 1, 9713 AV Groningen, The Netherlands; a.alipour.najmi.iranag@rug.nl (A.A.N.); r.p.h.bischoff@rug.nl (R.B.)

**Keywords:** *N*-dealkylation, opiate alkaloids, drug metabolite, tropane alkaloids

## Abstract

*N*-dealkylation, the removal of an *N*-alkyl group from an amine, is an important chemical transformation which provides routes for the synthesis of a wide range of pharmaceuticals, agrochemicals, bulk and fine chemicals. *N*-dealkylation of amines is also an important in vivo metabolic pathway in the metabolism of xenobiotics. Identification and synthesis of drug metabolites such as *N*-dealkylated metabolites are necessary throughout all phases of drug development studies. In this review, different approaches for the *N*-dealkylation of amines including chemical, catalytic, electrochemical, photochemical and enzymatic methods will be discussed.

## 1. Introduction

The carbon-nitrogen bond is ubiquitous in organic molecules and biomolecules and is well represented in all proteins as amino acids and in nucleic acids as nitrogenous bases. The amine functional group that has one or more C-N bonds is also present in large numbers of bulk chemicals, fine chemicals, agrochemicals and pharmaceuticals [1,2]. More than half of the top 200 small molecule pharmaceuticals by retail sales in 2020 contain an amine functional group in part of their chemical structure, covering a broad spectrum of therapeutic applications [3]. The basicity and electronic characteristics of the amines in these therapeutics generally provide positively charged entities which are crucial for interactions with the target receptors and are prone to oxidation processes with enzymes responsible for the metabolism of drugs [4]. Transformations of C-N bonds into different functional groups have been studied extensively in biochemistry, organic chemistry and electrochemistry [2,5,6]. 

Among the different transformations, cleavage of the C-N bond is an important in vivo metabolic reaction of drugs containing amines, catalyzed by members of the cytochrome P450 family of enzymes [7]. Several studies have investigated the mechanistic properties of the enzyme-catalyzed *N*-dealkylation reaction and researchers tried to simulate specific P450-catalyzed *N*-dealkylation reactions using electrochemistry and enzyme-derived systems (Section 5 and Section 6). Another important *N*-dealkylation reaction in living systems is the repair of alkylated DNA, which eliminates the alkylation damage at nitrogen atoms in nucleobases in the DNA structure upon the exposure of living cells to 3alkylating agents. If the *N*-alkylated modifications in the DNA structure are left unrepaired by the MGMT and ALKBH family of proteins, they compromise genomic integrity and disrupt different processes in living cells such as replication and/or initiate apoptosis [8].

Besides its importance in in vivo reactions, *N*-dealkylation of amines is a valuable synthetic tool for the synthesis of different agrochemicals, pharmaceuticals and fine chemicals. For example, *N*-demethylation of opiate alkaloids to their secondary amine derivatives is an important chemical step in the semi-synthesis of a wide range of opioid medicines. However, the high dissociation energy of C-N bonds in general and the stability of C-N bonds in amines in particular, have made their cleavage a great challenge in synthetic organic chemistry [2]. Especially demethylation reactions have been identified as a key green chemistry research area by pharmaceutical companies, which requires the development of alternative routes [9].

Several comprehensive reviews have summarized strategies for C-N bond activation and cleavage in a wide variety of nitrogen-containing chemical structures [2,10]. In this review, only C-N bond cleavage in amines in the context of *N*-dealkylations will be discussed. We will cover the *N*-dealkylation of amines, for which the newly synthesized *N*-dealkylated amines are the main product of the reaction and constitute added-value chemicals. 

## 2. General Chemical Methods for *N*-Dealkylation of Amines

Chemical methods are commonly used for the *N*-dealkylation of tertiary amines in natural products allowing re-alkylation and derivatization of the corresponding secondary amines. An important example for this is the *N*-demethylation of opiate alkaloids using different synthetic methods, a topic that will be discussed throughout all sections of this review. 

**Figure 1:**
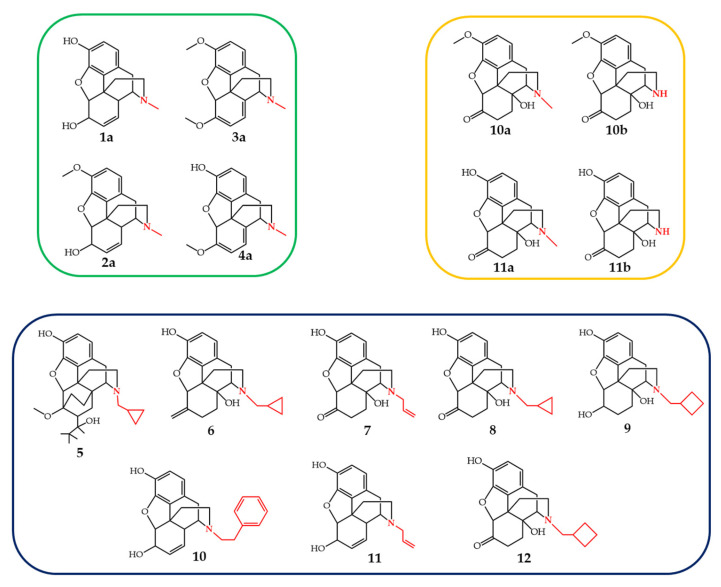
Naturally occurring opiate alkaloids (**green box**), opiate intermediates (**yellow box**), and opiate pharmaceuticals (**blue box**).

All opioid medicines in use today are semi-synthesized from naturally occurring opiates such as morphine **1a**, codeine **2a**, oripavine **3a** and thebaine **4a**. Buprenorphine **5** is an opiate agonist and is used as analgesic to reduce moderate to severe pain but its primary use is to treat opioid dependence. Nalmefene **6** and Naltrexone **7** are used for the treatment of patients with addiction and dependence on alcohol or opiates. Naloxone **8** is an opiate antagonist listed on the Model List of Essential Medicines established by the World Health Organization and is used in life-threatening situations arising from synthetic or natural opiate overdose. Nalbuphine **9** is a mixed opiate agonist-antagonist which is used as analgesic to avoid undesirable effects of morphine [11,12]. The chemical structures of various semi-synthesized opioid medicines are given in Figure 1 (blue box). The transformation of these natural compounds to pharmaceutically active agents requires different chemical steps among which *N*-demethylation is the most important and challenging step. After *N*-demethylation of the tertiary amine, the synthesized noropiates are functionalized with appropriate chemical groups to obtain specific therapeutic properties. A typical route for the synthesis of opioid medicines is the conversion of natural opiates **3**–**4** to oxycodone **10a** or oxymorphone **11a** followed by *N*-demethylation to noroxycodone **10b** or noroxymorphone **11b** and subsequent re-alkylation [12,13,14]. Therefore, different organic chemistry methods were developed for the selective *N*-demethylation of opiate alkaloids over a period of one century. 

### 2.1. N-Dealkylation of Amines by the von Braun Reaction

**Figure 2:**
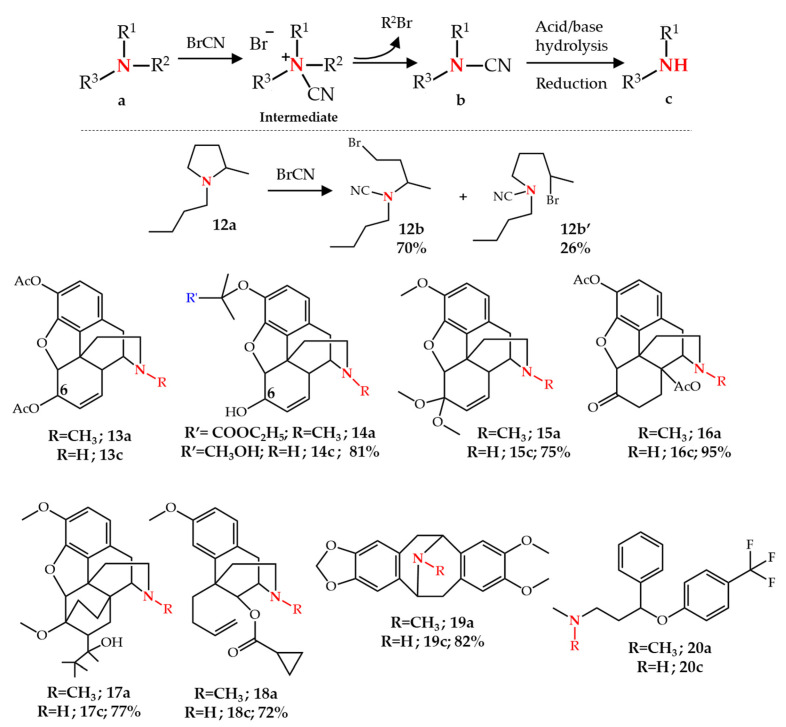
*N*-Dealkylation of amines by the von Braun reaction.

Von Braun reported his method for *N*-dealkylation of amines in 1900 and it is considered one of the oldest reported methods still used by synthetic chemists [15,16,17]. The general scope and mechanism of this reaction [18,19] and its application to *N*-demethylation of alkaloids [20,21] have been reviewed in detail. Generally, reaction of a tertiary amine with cyanogen bromide in an inert solvent such as chloroform or ether leads to the bromide salt of a quaternary cyanoammonium intermediate, which is then converted to a cyanamide and an alkyl bromide. Subsequent acid- or base-hydrolysis [22,23,24,25] or reduction [26,27] of the cyanamide leads to the desired secondary amine. Although acyclic amines in the von Braun reaction are converted to two distinct cyanamides and alkyl bromides, cyclic amines may undergo ring-opening resulting in the formation of a terminal long-chain bromo-alkyl-cyanamide, as shown for the conversion of compound **12a** to **12b** and **12b′**. However, normally the removal of the methyl group attached to the ring nitrogen is preferred to the ring opening [19,28] (Figure 2).

This method is broadly used for the *N*-demethylation of natural compounds. Generally, the phenol moieties in the morphinan structure need to be protected, as shown for heroin **13a**, before treatment with cyanogen bromide and subsequent hydrolysis [23,25]. However, the reaction can be performed without the protection of the C6-hydroxyl group of morphine in good yields, for example in compound **14** [26]. Rapoport et al. [27] reported the *N*-demethylation of codeinone dimethyl ketal **15a** using BrCN and subsequent reduction using LiAlH_4_ in 75% yield. This method was also used for the *N*-demethylation of 3,14-diacetyloxymorphone **16a** to **16c** in 95% yield using 25% aqueous H_2_SO_4_ in the hydrolysis step [29]. Other examples of *N*-demethylation of opiates include the *N*-demethylation of the β-thevinone derivative **17** in 77% yield [30] and the benzomorphan derivative **18** in 72% yield [31] (Figure 2). This method was recently used in the multi-step conversion of thebaine **4** to noroxymorphone **11b** [32] and buprenorphine **5** on an industrial scale [33].

Besides opiate alkaloids this reaction was also applied to other classes of molecules [19]. For example, eschscholtzidine **19a** (a pavine alkaloid) was converted to its corresponding cyanamide upon treatment with BrCN in 80% yield. Subsequent hydrolysis of the cyanamide under basic conditions led to noreschscholtzidine **19c** in 82% yield [34]. Fluoxetine **20c**, an antidepressant registered in the WHO list of essential medicines, can also be synthesized from **20a** following a one-step *N*-demethylation using the von Braun method [35] (Figure 2). 

Limited selectivity of this reaction due to the high reactivity of BrCN and its considerable toxicity are the major drawbacks which limit its application. Therefore, new methods based on chloroformates were developed which are discussed in the next section.

### 2.2. N-Dealkylation of Amines by Chloroformates

**Figure 3:**
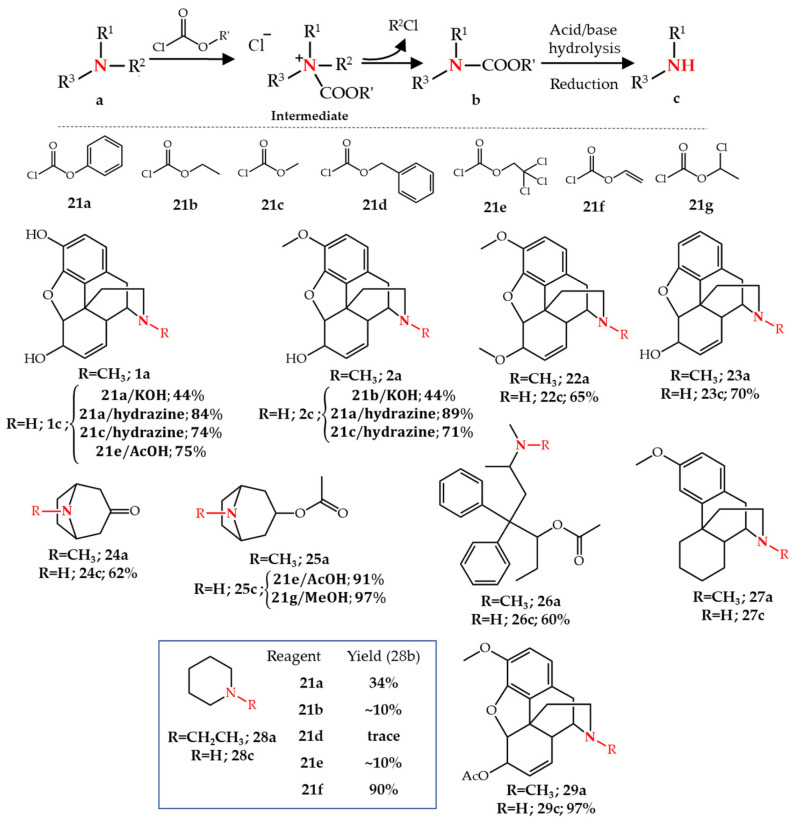
*N*-Dealkylation of amines by chloroformates.

Numerous studies have reported the utilization of different types of chloroformate reagents **21** (also known as carbonochloridates) for the *N*-dealkylation of tertiary amines. Their application to amines in general [36,37] and natural compounds in particular [20,21] has been reviewed in detail. Therefore, only several important examples will be discussed in this section. Generally, a tertiary amine reaction with a chloroformate reagent leads to a carbamate and an alkyl chloride via the formation of the chloride salt of a quaternary ammonium species. Subsequent hydrolysis of the carbamate yields the desired secondary amine [20] (Figure 3).

This method is broadly applied to the *N*-demethylation of alkaloids. For example, phenyl and ethyl chloroformate **21a**–**b** were used for the *N*-demethylation of morphine **1a** and codeine **2a**, respectively, both in 44% yield. A base (such as KHCO_3_ or KOH) was required in the presence of the chloroformate for this reaction, presumably for deprotonation and activation of the basic amine in the opiate. The carbamate intermediate needed to be isolated prior to the hydrolysis step, which required vigorous conditions of 50% aqueous or ethanolic KOH for the hydrolysis of the carbamate intermediate [38]. Milder and more effective conditions using hydrazine in the hydrolysis step were developed by Rice [39,40] in which phenyl chloroformate **21a** in the presence of sodium or potassium bicarbonate were used in the first reaction step. Moreover, the crude carbamate intermediate was used directly in the hydrolysis step. An excellent yield of normorphine **1c** (84%) and norcodeine **2c** (89%) was obtained by this method. Later, Brine et al. [41] showed that the substitution of phenyl chloroformate by methyl chloroformate **21c** in Rice’s procedure led to slightly lower yields of normorphine (74%) and norcodeine (71%), but purification of the final crude mixture was easier, since removing phenol as a byproduct is harder than the more volatile methanol that is released upon the hydrolysis of the carbamate. In other studies, a codeine derivative **22c** was obtained with the phenyl chloroformate/hydrazine approach in 65% yield [42] while the methyl chloroformate/hydrazine approach was applied for the *N*-demethylation of morphine derivative **23a** in 70% yield [43] (Figure 3). 

As an alternative to alkyl/phenyl chloroformate, 2,2,2-trichloroethyl chloroformate **21e** was first introduced for the *N*-demethylation of tropanes **24**–**25** [44]. This reagent allowed the facile conversion of the carbamate intermediate to a secondary amine upon treatment in acetic acid or methanol in the presence of zinc. Besides tropanes, the opiates noracetylmethadol **26c** and normorphine **1c** were obtained in 60% and 75% yield, respectively [44]. Other studies used this procedure for the *N*-demethylation of dextromethorphan **27a** [45,46] (Figure 3). The cleavage of the carbamate intermediate obtained by vinyl chloroformate **21f** was found to be more effective than the previously reported chloroformates. Using *N*-ethylpiperidine **28a** with chloroformates **21a**,**b**,**d**,**e**,**f**, Olofson et al. [47] showed that the carbamate intermediate of **28a** was obtained in 90% yield with **21f** compared to 10–34% with chloroformates **21a**,**b**,**d**,**e**. Hydrolysis of the carbamate intermediates in aqueous HCl led to the HCl salt of the corresponding secondary amines. The authors proposed that the improved selectivity of **21f** compared to other chloroformates is related to an increased electrophilicity at the acyl carbon adjacent to the electron withdrawing vinyl group as well as to the steric factors. The authors showed that this method is very useful for the *N*-demethylation of **16a** following carbamate intermediate formation and hydrolysis of carbamate and acetyl groups to directly obtain **11b** in 98% crude yield [47]. Other studies also reported the successful application of this method for the *N*-demethylation of various opiate alkaloids [48,49] (Figure 3).

α-Chloroethyl chloroformate **21g** was used for the selective *N*-demethylation of tertiary amines including alkaloids with a facile hydrolysis step. Upon treatment of *N*-ethylpiperidine **28a** with **21g** in ethylene chloride, the carbamate intermediate was formed which was used without further isolation in the next hydrolysis step. The removal of solvent followed by dissolution in methanol at 50 °C resulted in an HCl salt of the secondary amine, without adding any HCl. Compared to vinyl chloroformate **21e**, α-chloroethyl chloroformate is cheaper and does not require any HCl to form the secondary amine salt in the second step. Other examples of this procedure include the *N*-demethylation of *O*-acetyltropine **25a** and 6-acetylcodeine **29a** both in 97% yield [50] (Figure 3).

**Figure 4:**
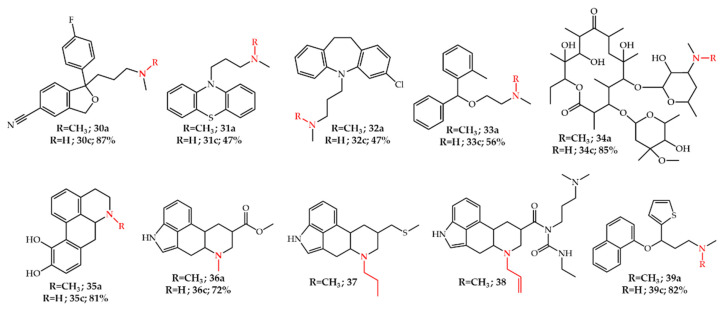
*N*-Dealkylated drug metabolite synthesis with chloroformates.

Besides natural tertiary amines, this method is also widely applied for the *N*-demethylation of fine chemicals. For example, the latter method based on **21g** was used for the synthesis of *N*-demethylated drug metabolites of citalopram **30a**, an antidepressant used for the treatment of anxiety, in 87% yield [51]. *N*-demethylated drug metabolites of promazine **31a**, clomipramine **32a**, orphenadrine **33a** [52], and erythromycin **34a** [53,54] were also synthesized using this procedure. Kim [55] reported the *N*-demethylation of apomorphine **35a** using phenyl chloroformate and subsequent reduction by hydrazine in 81% yield. The method was also used for the *N*-demethylation of ergolines (ergot alkaloids) with 2,2,2-trichloroethyl chloroformate in the presence of potassium bicarbonate as a base to obtain a carbamate derivative of ergoline **36** in 90% yield. This intermediate was hydrolyzed in acetic acid in the presence of Zn powder at room temperature to afford the secondary amine **36c** in 72% isolated yield [56]. Recently, this method was used for the synthesis of the Parkinson’s disease medicines pergolide **37** and cabergoline **38**, on an industrial scale [57]. **37** and **38** can be synthesized via *N*-demethylation of **36a** and subsequent functionalization of secondary amine **36c**. The main difference in this study was the application of only 5 mol% 4-(*N*,*N*-dimethylamino)pyridine (DMAP) as base [57] instead of 5 equivalents of KHCO_3_ [56] leading to a considerable decrease in the solid waste while increasing the purity and isolated yield of the carbamate intermediate of **36** to 94% in kg scale (Figure 4).

Duloxetine **39c** is an FDA approved therapeutic drug which is used for the treatment of depressive disorders [58]. **39c** can be synthesized by *N*-demethylation of **39a** which in turn is synthesized in a multi-step procedure. Deeter et al. [59] reported the application of **21e** followed by a hydrolysis step with zinc dust in formic acid for the *N*-demethylation of **39a** in 82% yield. A process based on this method was also patented for the synthesis of fluoxetine **20c** via *N*-demethylation of **20a** [60] (Figure 4).

## 3. Transition-Metal Catalyzed *N*-Dealkylation

### 3.1. Palladium-Catalyzed

Among the different transition-metal catalyzed *N*-dealkylation methods, Pd-catalyzed *N*-dealkylation of amines is one of the most widely studied and best developed strategies for the synthesis of pharmaceutical intermediates and therapeutics in small to large scales.

The recent annual reports of the International Narcotics Control Board (INCB) showed that the global manufacture of buprenorphine **5** was 17.2 and 10.5 tons in 2018 and 2019, respectively. The estimated annual needs for oxymorphone **11a**, noroxymorphone **11b** and oxycodone **10a**, for conversion purposes, in the United States for 2021 were 28.2, 22 and, 57 tons, respectively; all three are potential starting compounds for the synthesis of opioid medicines [61,62,63]. Such a high demand and consequent large-scale production of these pharmaceuticals requires sustainable, efficient and scalable *N*-demethylation methods, and the Pd-catalyzed *N*-demethylation of opiate alkaloids is the most developed strategy, which has been put into practice for different opiate alkaloids in large scale.

**Figure 5:**
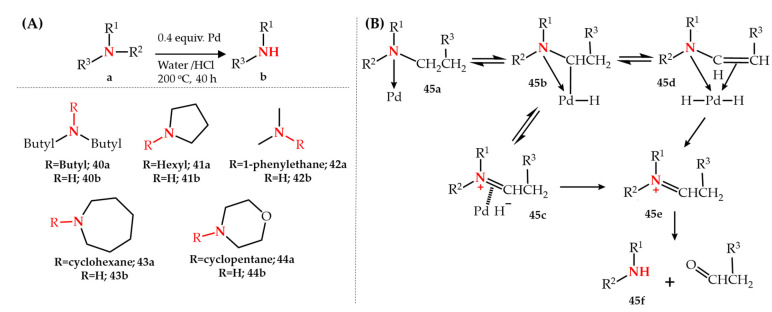
(**A**) Pd-catalyzed *N*-dealkylation of aliphatic and cyclic tertiary amines and (**B**) plausible mechanistic pathways.

The first study of Pd-catalyzed *N*-dealkylation of amines was introduced by Murahashi et al. [64] in 1979 using palladium black as catalyst in the presence of hydrochloric acid for the catalytic hydrolysis of a variety of aliphatic and cyclic tertiary amines **40a**–**44a**. The reaction was performed in water as the only solvent at a temperature of 200 °C with a catalyst-to-amine ratio of 40% and an HCl-to-amine ratio of 35%. The authors reported that the application of other palladium compounds such as PdCl_2_ or Pd(OAc)_2_ as catalyst gave similar results. *N*-dealkylation of different alkyl groups showed that the cleavage of the C-N bond is easier in the order of methine > methylene > methyl. The reaction is capable of removing aliphatic groups such as butyl and hexyl and cyclic groups such as cyclohexane, cyclopentane. A plausible mechanistic pathway was presented which included an initial coordination of the lone pair electrons of nitrogen to palladium (**45a**) followed by palladium insertion into the adjacent C-H bond (**45b**) which is in equilibrium with complexes of **45c** and **45d**. Upon protonolysis of the intermediate complexes, an iminium ion (**45e**) is formed which hydrolyses to the secondary amine **45f** and the corresponding carbonyl compounds (Figure 5).

**Figure 6:**
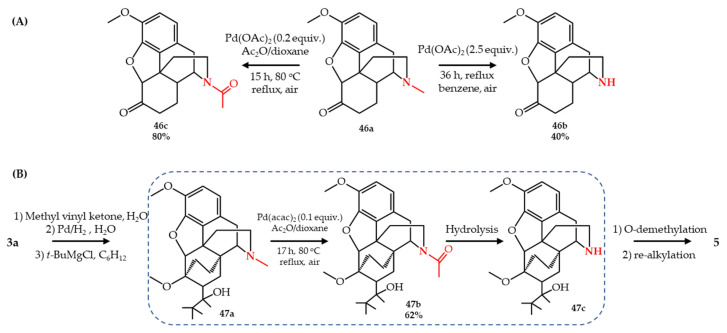
Pd-catalyzed *N*-demethylation/*N*-acylation of opiates of (**A**) hydrocodone (**46a**) and (**B**) oripavine (**3a**).

The first report of palladium-catalyzed *N*-demethylation of opiates showed the conversion of hydrocodone **46a** to norhydrocodone **46b** [65]. A 2.5 equivalent of palladium acetate was used as catalyst in heated benzene and refluxed to obtain **46b** in 40% yield with 55% recovery of starting **46a**. It was noted that this reaction only occurred for **46a** and failed when applied to other opiates such as oxycodone **10a**, morphine **1a** or codeine **2a**. The same reaction in the presence of acetic anhydride led to concurrent *N*-demethylation/*N*-acylation of hydrocodone, as an alternative 2-step *N*-demethylation strategy. A 0.2 equivalent of palladium acetate in dioxane/acetic anhydride was heated to 80 °C and refluxed to obtain *N*-acetylnorhydrocodone **46c** in 80% yield, which can be hydrolyzed to **46a** (Figure 6A). This methodology was later used for the semi-synthesis of buprenorphine **5** from thebaine **3** by Machara et al. [66]. A key advanced intermediate **47a** obtained from **3a** was used as starting compound which concurrently *N*-demethylated/*N*-acylated to **47b** with 62% yield using either Pd(OAc)_2_ (palladium(II) acetate) or Pd(acac)_2_ (palladium(II) acetylacetonate). The base hydrolysis of the *N*-acetyl bond forming the secondary amine derivative **47c**, followed by re-alkylation and subsequent O-demethylation led to buprenorphine **5** with an overall 65% yield (Figure 6B). 

**Figure 7:**
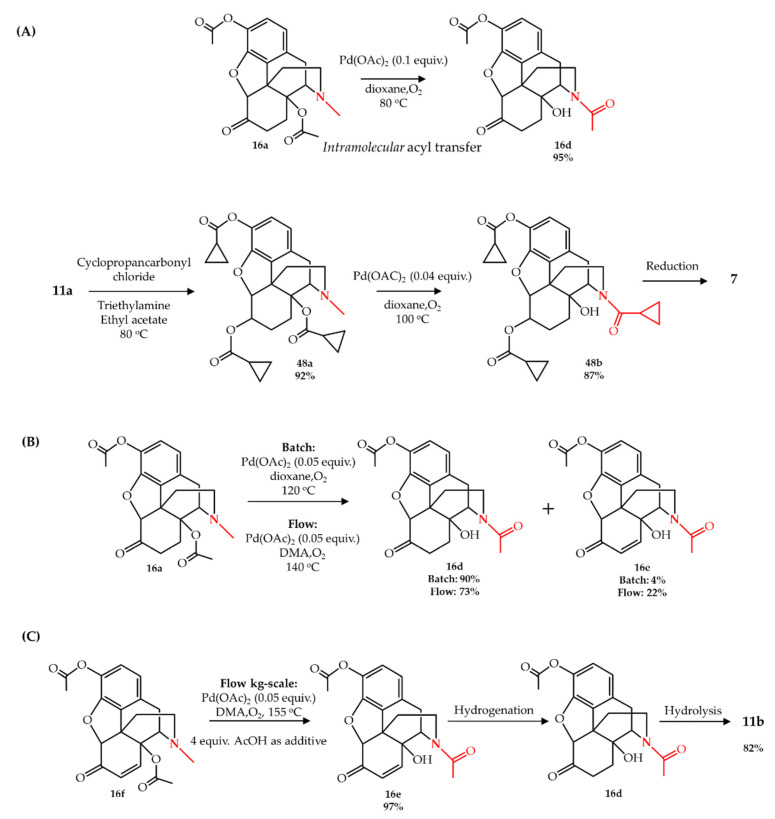
Pd-catalyzed intramolecular acyl transfer in opiates: (**A**) conversion of 3,14-diacetate oxymorphone (**16a)** according to Machara et al. [67], (**B**) conversion of **16a** according to Gutmann et al. [68], and (**C**) conversion of **16f**, the hydrogenated form of **16a**, according to Gutmann et al. [68].

Following their previous study, Machara et al. [67] used a conceptually similar approach for the synthesis of naltrexone **7** using *N*-demethylation/*N*-acylation of a specific derivative of oxymorphone, 3,14-diacetate oxymorphone **16a**. The major difference was that instead of *N*-demethylation/*N*-acylation of the *N*-methyl moiety using acetic anhydride as acetyl source, an intramolecular acyl transfer occurred converting bis-*O*-acetyloxymorphone to its *N*-acetyl derivative **16d**, using Pd(OAc)_2_ as catalyst. The other difference is the application of pure oxygen as oxidant instead of air. Following this interesting observation, an intermediate compound **48a** obtained from oxymorphone **11a** was used as a starting compound for the semi-synthesis of **7** which was *N*-demethylated/*N*-acylated to **48b** followed by one-step reduction to obtain **7** (Figure 7A).

Gutmann et al. [68] used the same intramolecular acyl transfer strategy for the synthesis of noroxymorphone **11b** on an industrial scale in a tubular flow reactor, using Pd(OAc)_2_ as catalyst and oxygen as oxidant at a temperature of 120–160 °C. Their preliminary results in a microwave reactor using starting compound **16a** showed that changing the reaction temperature from 80 °C [67] to 120 °C considerably increased the rate of reaction from about 20 h [67] to less than 2 h, in small-scale synthesis. Moreover, the reaction proceeds in DMA (dimethylacetamide) as solvent as well as in dioxane. However, in every condition they observed the formation of **16e** as byproduct, which is the dehydrogenated form of **16d**. Continuous flow conversion of **16a** to **16d** also gave 22% of **16e** under optimal conditions beside the main acyl-transferred product (Figure 7B). In order to avoid the formation of the byproduct, they changed the starting compound from **16a** to its precursor compound **16f** (**16f** is converted to **16a** by a one-step hydrogenation). Continuous flow *N*-demethylation/*N*-acylation of **16f** led to 93% of **16e** with less than 1% of other byproducts, in small scale. Finally, they converted **16f** to **16e** in kg-scale with 97% yield and only 3% of other byproducts in a sophisticated flow system. A flow-hydrogenation of **16e** followed by a batch-hydrolysis using sulfuric acid in an *n*-BuOH/H_2_O solvent system led to ~80% isolated yield of noroxymorphone **11b** (Figure 7C).

**Figure 8:**
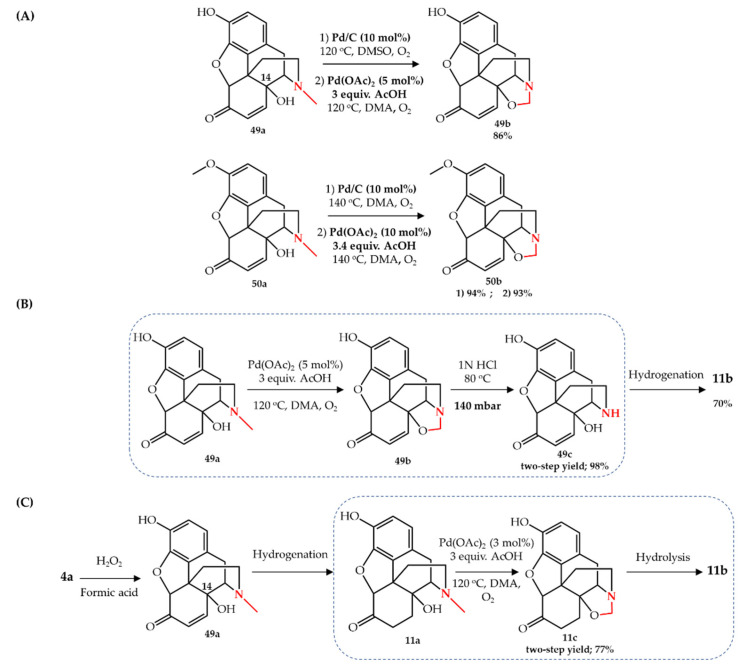
Pd-catalyzed oxazolidination of opiates: (**A**) 14-hydroxymorphinone **49a** and 14-hydroxycodeinone **50a** according to Gutmann et al. [69], (**B**) **49a** according to Gutmann et al. [69]**,** and (**C**) oxymorphone **11a** according to Gutmann et al. [13].

Gutmann et al. [69] later reported a two-step *N*-demethylation strategy for the synthesis of noroxycodone **10b** and noroxymorphone **11b** using Pd/C as catalyst and oxygen as terminal oxidant at a temperature of 120–140 °C. They reported the formation of an oxazolidine intermediate **49b**–**50b** upon the catalytic oxidation of 14-hydroxymorphinone **49a** or 14-hydroxycodeinone **50a** (Figure 8A). The oxazolidine structure can be readily acid hydrolyzed to the corresponding nor-form which then only requires one more hydrogenation step to afford **10b** or **11b**. A broad screening of different solvents showed that the palladium-catalyzed oxazolidine formation only works in DMA or DMSO (less than 5% yield was achieved in NMP, MeCN, EtOAc, dioxane, toluene, *i*-PrOH, AcOH, and butanone). The formation and presence of formaldehyde during the acid hydrolysis of the oxazolidine structure was found to be detrimental for the selectivity of the hydrolysis, as different unidentified byproducts were observed in this step. In order to remove the formaldehyde from the system, the hydrolysis step was carried out at lower pressure (140 mbar) and 80 °C, leading to a high selectivity of 90% in a very short time (<5 min). Moreover, they reported the in situ formation of Pd(0) colloid particles as catalyst instead of Pd/C. The heating of a mixture of palladium acetate in the presence of acetic acid in DMA to 120–140 °C led to the formation of a deep-dark solution of finely dispersed Pd(0) particles which has the same catalytic efficiency as Pd/C toward the formation of **49b** and **50b**. Indeed, they reported the application of AcOH as additive in their previous kg-scale synthesis of noroxymorphone [68]. A one-pot two-step gram-scale conversion of **49a** to **49c** using in situ formation of catalyst was then carried out with 98% yield after the two steps. Subsequent hydrogenation of **49b** under flow conditions led to noroxymorphone **11b** with 70% overall yield (Figure 8B).

Following their previous study, Gutmann et al. [13] designed and developed a continuous flow system for the conversion of oxymorphone **11a** to noroxymorphone **11b** through the oxazolidine intermediate. They also started the reaction with **49a**, but switched the hydrogenation reaction step to be the first step (to obtain **11a**) and used the palladium-catalyzed oxidation of *N*-methyl group as the second step, before hydrolyzing the oxazolidine structure to produce noroxymorphone (Figure 8C). As they previously reported [69], a Pd(0) colloid was freshly prepared using palladium acetate and acetic acid before starting the reaction and was used both for the hydrogenation and oxazolidine formation reactions. Processing the crude product of the hydrogenation reaction through an aerobic oxidation reaction showed that the initial colloidal Pd(0) catalyst can be utilized both for the hydrogenation and the subsequent oxidation step. However, due to the low activity of the Pd(0) catalyst in the second step, and the therefore diminished selectivity of oxazolidine formation (80–90%), a freshly prepared Pd(0) catalyst was used in the second step. A 77% isolated yield of the oxazolidine **11c** was obtained through crystallization upon concentration of the final crude mixture and addition of cold water. The synthesized oxazolidine was hydrolyzed to **11b** using their previously reported hydrolysis method [69]. A very similar approach for the continuous flow synthesis of **11b** from **49a** was also reported [14]. The difference in this study was that continuous C-14 hydroxylation of oripavine **4a** (as the natural starting compound) was used to obtain advanced intermediate **49a** in the flow condition and a commercially available Pd/C flow cartridge was used for the hydrogenation step. Palladium-catalyzed aerobic oxidation of **49a** was performed under the same optimal conditions as reported in [13] leading to the oxazolidine **11c** with similar final yields. 

### 3.2. Iron-Catalyzed

**Figure 9:**
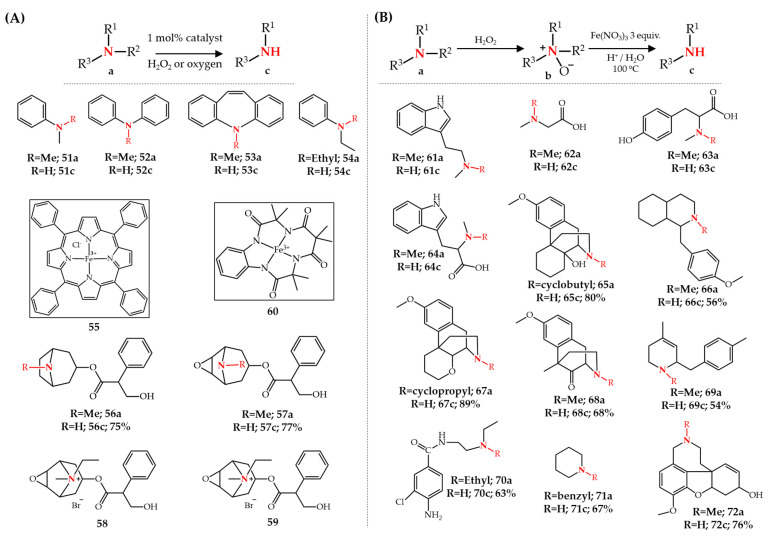
Fe-catalyzed *N*-dealkylation of tertiary amines (**A**) without and (**B**) with *N*-oxide formation.

In efforts to mimic the cytochrome P-450-catalyzed *N*-dealkylation transformation and understand its mechanistic pathways in living organisms, various model reactions have been presented for the oxidation of amines, investigating the application of iron salts as catalysts such as FeCl_3_ [70], Fe(ClO_4_)_3_ [71], [Fe(II)(MeCN)_4_](ClO_4_)_2_ [72], and iron porphyrins [73,74] using oxygen [70,71,75], hydrogen peroxide [72], *tert*-butyl-hydroperoxide or iodosyl benzene [74], as oxidants. For example, Santa et al. [73] reported the *N*-dealkylation of various tertiary amines **51**–**54** at the analytical scale using an iron porphyrin, Fe(III)TPPCl **55**, as catalyst in the presence of O_2_ as oxidant. The reaction proceeds at room temperature in a protic solvent system of CH_2_Cl_2_–MeOH–H_2_O (3:6:1) with a very low catalyst loading of 1 mol%, yielding secondary amines (Figure 9A).

Recently, Do Pham et al. [76] used this strategy for the oxidative *N*-demethylation of the natural tropane alkaloids atropine **56a** and scopolamine **57a** in preparative scales with 70–80% yield. Noratropine **56c** and norscopolamine **57c** are key intermediates for the semi-synthesis of the important bronchodilator medicines ipratropium bromide **58** and oxitropium bromide **59**, the former of which is registered on the essential medicine list of WHO. They used Fe(III)-TAML **60** as iron catalyst and hydrogen peroxide as oxidant in a one-pot *N*-demethylation strategy in which 50 equivalents of H_2_O_2_ were added in small portions to the reaction mixture. Excess hydrogen peroxide needed to be removed by adding MnO_2_ at the end of the reaction. The reaction works at room temperature in non-hazardous solvents such as ethanol and only 1 mol% of catalyst. An important result of this reaction was that the final reaction mixture is very clean and the synthesized nortropane alkaloids can be isolated with high purity by convenient liquid-liquid extraction without any need for chromatographic purification. Their effort to expand this catalytic synthetic tool to opiate alkaloids was not successful. The Fe(III)-TAML-catalyzed oxidative *N*-demethylation of thebaine **3a** in the presence of H_2_O_2_ did not lead to any northebaine **3b** as product while the same reaction for oxycodone **10a** only resulted in 17% yield of noroxycodone **10b** [77] (Figure 9A). 

A modified version of the iron-oxidant catalytic system, known as the modified-Polonovski reaction or non-classical-Polonovski reaction, was developed to study the role of amine *N*-oxide formation during the *N*-dealkylation in cytochrome P450-catalyzed transformations. Polonovski discovered that the treatment of a tertiary amine *N*-oxide with acetyl chloride or acetic anhydride leads to the cleavage of one of the *N*-alkyl groups producing the *N*-acetyl derivatives of the corresponding secondary amine and an aldehyde [78]. In the modified Polonovski reaction, the acetyl chloride or acetic anhydride is replaced by an iron-containing reagent while a tertiary amine *N*-oxide is prepared by direct oxidation of the tertiary amine with an oxidant such as hydrogen peroxide. Horning et al. [79,80] first discovered that the treatment of *N*,*N*-dimethyltryptamine *N*-oxide **61b** in acidic aqueous solution at elevated temperature in the presence of Fe^3+^ ions resulted in *N*-methyltryptamine **61c** as product. Three equivalents of Fe(NO_3_)_3_ in the presence of oxalic acid in water at a temperature of 100 °C led to about 50% *N*-demethylation of **61a** to **61c**. Importantly, they reported that the replacement of iron with other metal ions such as cobalt(II), nickel(II), zinc(II), magnesium(II), manganese(II), and copper(II) did not lead to any *N*-demethylation reaction, supporting the critical role of iron ions in this reaction. Horning et al. later successfully used iron(III) for the *N*-demethylation reaction on other tertiary amine structures such as *N*,*N*-dimethylglycine *N*-oxide **62b** [81], *N*,*N*-dimethyltyrosine *N*-oxide **63b** and *N*,*N*,dimethyltryptophan *N*-oxide **64b** [82] (Figure 9B). 

Monkovic et al. [83] later developed and used a modified-Polonovski reaction for the synthetic application to produce different fine chemicals through *N*-dealkylation of various tertiary amine *N*-oxides including important morphinan structures **65a**–**71a**. The reported one-pot, two-step *N*-dealkylation method consists of the addition of *m*-chloroperbenzoic acid (*m*-CPBA) to a tertiary amine in dichloromethane followed by the addition of aqueous iron (II) chloride solution (40 mol% FeCl_2_) at a temperature of −10 °C–0 °C leading to high yields of secondary amines. Mary et al. [84] used the same strategy for the selective *N*-demethylation of galanthamine **72a** to norgalanthamine **72c** with 76% isolated yield. The main difference in their method was the application of FeSO_4_ as catalyst in methanol at a temperature of 10 °C (Figure 9B).

**Figure 10:**
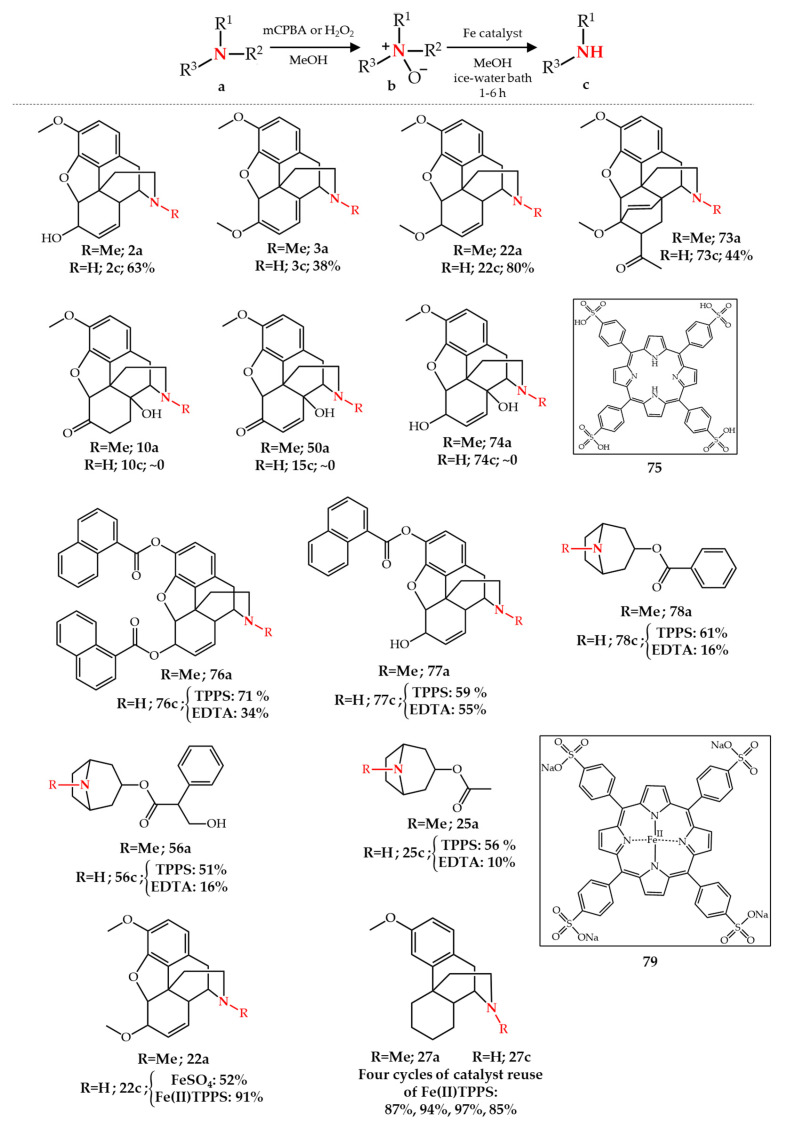
Fe-catalyzed *N*-demethylation of opiate and tropane alkaloids via *N*-oxide formation.

Following these first reports, numerous studies applied the modified-Polonovski reaction for the *N*-demethylation of opiate and tropane alkaloids. In an early study McCamley et al. [85] reported the *N*-demethylation of various opiate alkaloids (**2a**, **3a**, **22a**, and **73a**) using H_2_O_2_ or *m*-CPBA for the formation of the corresponding *N*-oxide (or using magnesium bis(monoperoxyphthalate) hexahydrate [86]) and then using different iron salts such as FeSO_4_, FeCl_3_, Fe(NH_4_SO_4_)_2_ as catalyst to produce the corresponding secondary amine, among which FeSO_4_ was found to be the most effective. The synthesized *N*-oxide intermediate can be used directly in the next step as free amine or isolated as HCl salt upon treatment with HCl aqueous solution, the latter of which was found to afford a higher yield. The major finding in their study was that the C14-hydroxyl group plays an important role in the iron-mediated Polonovski reaction. None of the three opiate examples having a -OH group adjacent to the *N*-methylamine group, **10a**, **50a**, and **74a**, underwent the *N*-demethylation reaction upon the treatment of the corresponding *N*-oxide with iron (II), resulting in a negligible final yield. Moreover, the major limitation of this method is the difficulty in separating the final nor-product from the iron salt which is used in stoichiometric amounts. To solve this drawback, they used ethylenediaminetetraacetic acid (EDTA) or TPPS **75** as iron chelating agent during the final work-up and purification. The application of TPPS in the work-up procedure remarkably increased the final isolated yield of noropiate alkaloids **76c**–**77c** and nortropane alkaloids **25c**, **56c**, and **78c**, compared to the EDTA work-up procedure [86] (Figure 10).

Inspired by TPPS as chelating agent, another form of the iron(II) ion in the form of an iron porphyrin, Fe(II)TPPS **79**, was also used as catalyst in the modified-Polonovski reaction for the *N*-demethylation of opiate alkaloids [87]. With the same amount of added iron catalyst, application of Fe(II)TPPS considerably increased the final isolated yield of **22c** compared to FeSO_4_ from 52% to 91%. The major finding in this study was the recyclability of the catalyst **79**. Nordextrometorphan **27c** was obtained in consistently high yields over four cycles of catalyst recovery and reuse. However, Fe(II) is prone to oxidation to Fe(III) during the multi-step synthesis of Fe(II)TPPS from TPPS and caution should be taken to minimize the exposure of **79** to air to prevent oxidation. In order to overcome this challenge, an improved process for the *N*-demethylation of opiate alkaloids was reported using **79** as catalyst in sodium acetate buffer (1 M, pH = 4) [88]. In this method, Fe(II)TPPS was synthesized from its precursor TPPS in acetate buffer and was used directly without further isolation in the *N*-demethylation of tertiary amine *N*-oxides. Furthermore, an important result taken from this study was, that by increasing the reaction temperature to 50–100 °C, the catalyst consumption decreased by one order of magnitude (20 mol% to 2 mol%) while keeping the final isolated yield unchanged for nordextrometorphan **27c** (Figure 10).

**Figure 11:**
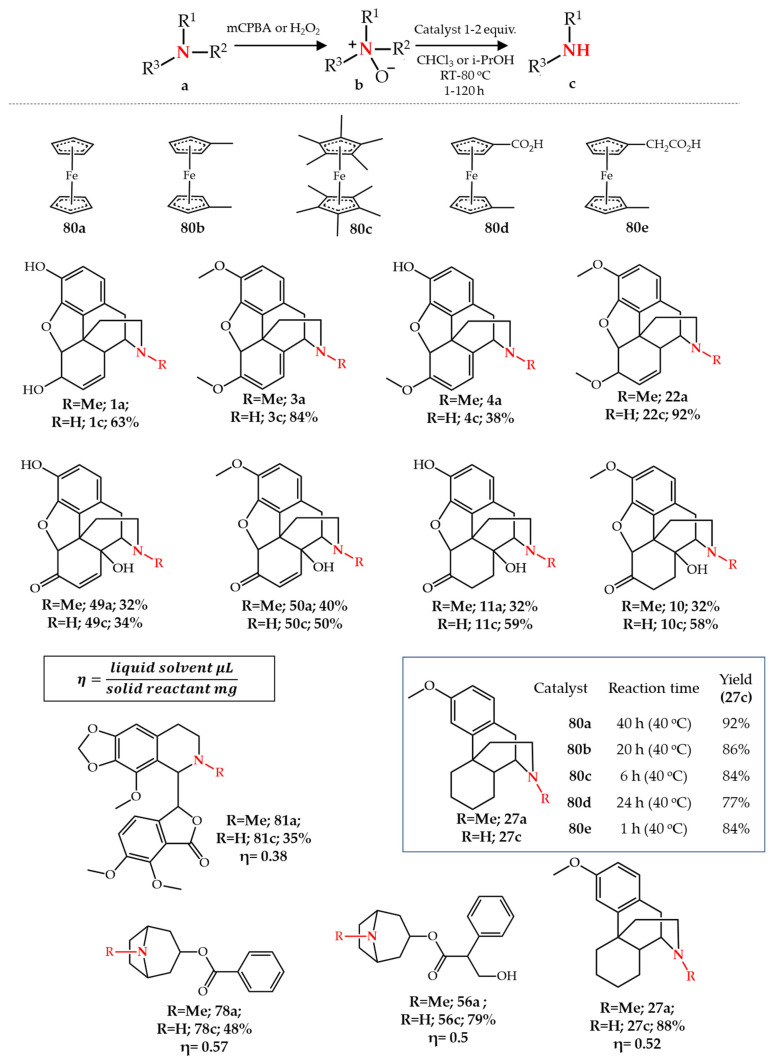
Fe-catalyzed *N-*demethylation of various alkaloids.

In the quest to find a better iron catalyst in the modified-Polonovski reaction, Kok et al. [89] used ferrocene **80a** for *N*-demethylation of a wide range of opiate and tropane *N*-oxides. In contrast to previous methods, this method can be applied to opiates with a C14-hydroxyl group such as **10**–**11**, and **49**–**50**. However, the final isolated yield is low and the corresponding *N*-methyl structure was also recovered during purification. Later, they investigated the effect of electron donor groups attached to ferrocene, catalysts **80a**–**e**, on *N*-demethylation of opiate *N*-oxides [90]. It was found that the rate of the reaction was enhanced by the increasing electron donor groups attached to the parent ferrocene while keeping the final isolated yield high (for example, **27c** was synthesized from **27a** using **80a**–**e** as catalyst). Kok et al. [91,92,93] subsequently studied the application of Fe(0) powder and stainless steel as substituents for the iron-catalyst which gave similar overall results. Their method using Fe(0) powder was also applicable to opiates with a hydroxyl group on carbon-14 yielding the desired noropiates structures in 40–60% yield [91]. Nakano et al. [94] later used this strategy in continuous flow conditions for the synthesis of **27c**. Application of zero-valent iron nanoparticles and Fe_3_(CO)_12_ as Fe(0) source in the modified-Polonovski reaction were also reported for *N*-demethylation of some opiate and tropane alkaloids [95] (Figure 11).

An interesting iron-based modified-Polonovski reaction using liquid assisted grinding (LAG) mechanochemistry was developed by Awalt et al. [96]. As organic solvents constitute 80–90% of nonaqueous waste produced in pharma industries, any attempt to reduce the consumption of solvent usage can drastically reduce the environmental footprint of chemical processes. LAG mechanochemistry allows us to perform chemical reactions in the presence of very low amounts of solvent. The reaction was carried out in a ball-milling closed vessel using Fe(0) dust as an iron catalyst in the presence of non-hazardous solvents such as ethanol and isopropanol, in which the ratio of liquid to solid reactant *(*η) in µL/mg is between 0 and 1. For regular chemical reactions, this ratio is more than 10. Besides tropanes and opiates, this method was also used for the *N*-demethylation of noscapine **81a**. Although the solvent usage in the *N*-demethylation stage is remarkably reduced, the final reaction mixture still requires chromatographic purification utilizing considerable amounts of solvent to obtain nor-compounds, while the *N*-oxide formation requires *m*-CPBA in chlorinated solvents [96] (Figure 11).

**Figure 12:**
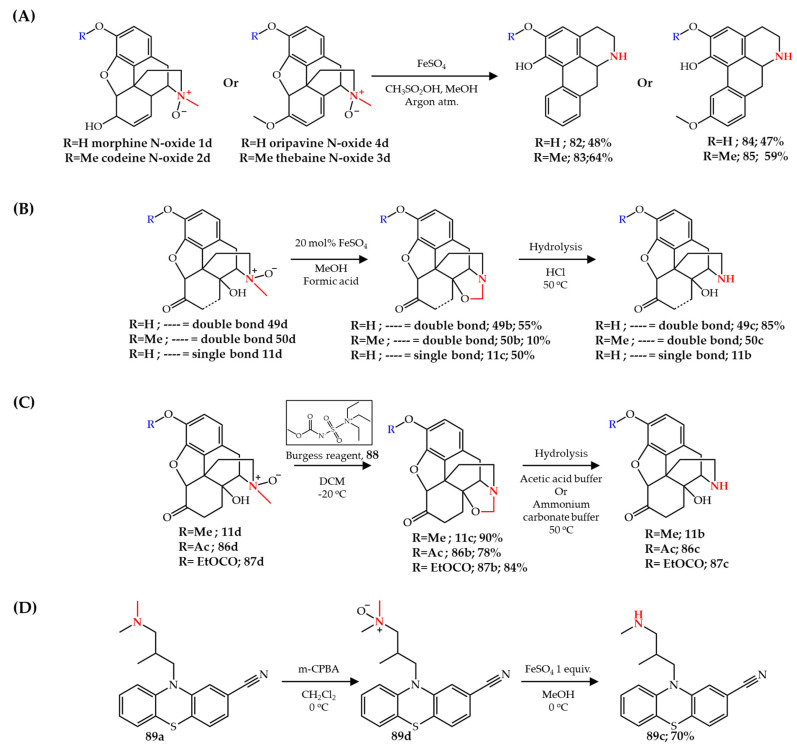
**(A**) Rearrangement and *N*-demethylation of aporphines, (**B**,**C**) oxazolidination of opiate *N*-oxides, and (**D**) *N*-demethylation of cyamemazine via *N*-oxide formation.

A one-pot *N*-demethylation and rearrangement of opiates **1**–**4** to noraporphines **82**–**85** (Figure 12), respectively, was also reported using iron-based *N*-demethylation of tertiary amine *N*-oxides [97]. The re-alkylation of noraporphines can lead to potentially serotonin- and/or dopamine-active compounds. In this method, opiate *N*-oxides were treated with FeSO_4_ in anhydrous methanol followed by methanesulfonic acid addition under argon atmosphere leading to methanesulfonic acid salts of the corresponding noraporphines via *N*-demethylation and rearrangement of the morphinan structure (Figure 12A).

In contrast to the well-developed Pd-catalyzed *N*-demethylation of 14-hydroxy opiates, only a limited number of studies have shown that the iron-catalyzed modified-Polonovski reaction is capable of *N*-demethylation of 14-hydroxy opiates and only in low yields [89,91]. Smith et al. [98] patented a different procedure for iron-catalyzed *N*-demethylation of 14-hydroxy opiates via oxazolidine formation, the same structure which is obtained by Pd-catalyzed *N*-demethylation strategies (see Section 3.1). However, in contrast to the Pd-catalyzed method which starts with a tertiary amine, a tertiary amine *N*-oxide served as a starting compound, similar to the Polonovski reaction. 14-hydroxy *N*-oxide opiates **11d**, **49d**, **50d** were converted to oxazolidine intermediates **11c**, **49b**, **50b** using FeSO_4_ as catalyst in the presence of formic acid. The oxazolidine intermediates can be hydrolyzed by a strong acid such as hydrochloric or sulfuric acid (Figure 12B).

Werner et al. [99] also reported the formation of the same oxazolidine intermediates from 14-hydroxy *N*-oxide opiates **11d**, **86d**, and **87d**. However, they used Burgess reagent **88** instead of iron-based reagents. They reported higher yields for oxazolidine formation compared to the procedure developed by Smith et al. [98]. Importantly, no *N*-demethylation reaction was observed upon the treatment of hydrocodone *N*-oxide with Burgess reagent confirming the importance of the C14 hydroxyl group for this reaction. The hydrolysis step in either acetic acid or ammonium carbonate buffer (pH = 9) at a temperature of 50 °C resulted in the corresponding secondary amine structure in high yields (Figure 12C). This strategy was later developed into a general method for the direct synthesis of opioid medicines with direct functionalization of oxazolidine intermediates without the oxazolidine hydrolysis step [100]. Besides the application of the iron-catalyzed reaction for the synthesis of pharmaceutical intermediates, it has also been used for the synthesis of drug metabolites [101,102]. For example, Singh et al. [101] used an FeSO_4_-mediated *N*-demethylation strategy for the synthesis of the *N*-demethylated metabolite of cyamemazine **89a**, a neuroleptic drug. A corresponding *N*-oxide **89d** was obtained using *m*-CPBA by Fe(II)-catalyzed *N*-demethylation to obtain **89c** in 70% overall yield (Figure 12D). 

### 3.3. Gold- and Platinum-Catalyzed

**Figure 13:**
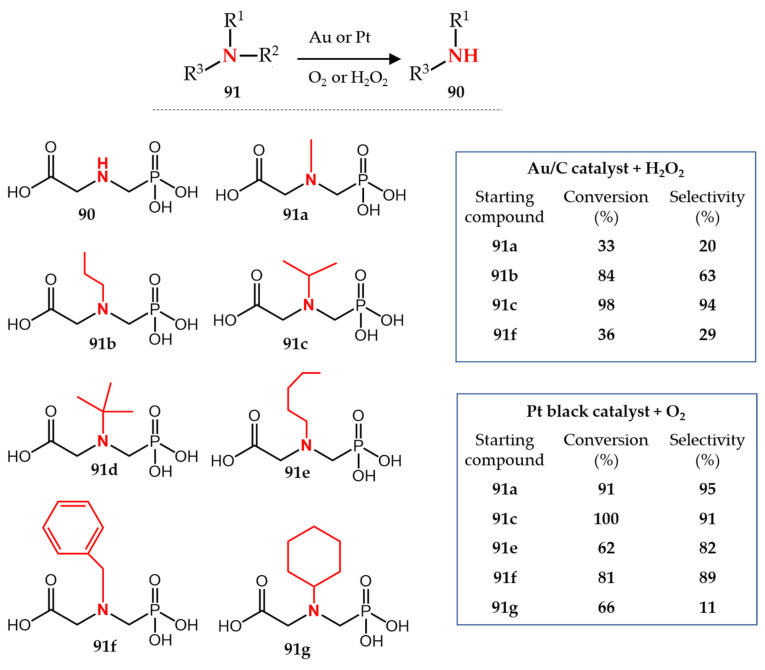
Glyphosate synthesis via Au- or Pt-catalyzed *N*-dealkylation.

Besides the application of the *N*-dealkylation reaction for the synthesis of pharmaceuticals and fine chemicals, this reaction can also be applied for the synthesis of bulk chemicals in megaton scale. Glyphosate **90** (*N*-phosphonomethyl glycine) is a major herbicide that is heavily used worldwide with a global consumption volume of more than 800 kiloton in 2014 and 1 megaton in 2017 [103,104]. Different industrial large-scale processes have been developed and usually patented for the synthesis of glyphosate since its commercialization in 1974 (Roundup, Monsanto Company, Manhattan, KS, USA), and those processes have recently been reviewed in detail [105]. Among the different multi-step synthesis procedures of glyphosate, an atom-efficient and eco-friendly procedure is the oxidative *N*-dealkylation of *N*-alkyl-substituted derivatives of *N*-phosphonomethyl glycine **91** as the final step (Figure 13).

Morgenstern et al. [106] reported a general strategy for the *N*-dealkylation of **91** using various supported and unsupported platinum catalysts, among which platinum black was the most efficient. The reaction proceeds with 30 wt% catalyst loading in water as solvent and under oxygen atmosphere at 80 °C. All starting *N*-substituted glyphosates (except for **91g**) were transformed to **90** with high conversion and selectivity. In a recent study, Yushchenko et al. [107] reported the application of carbon-supported gold nanoparticles for the efficient and selective *N*-dealkylation of *N*-isopropyl glyphosate **91a**. Compared to 30 wt% Pt catalyst, only 2 wt% of Au/C catalyst was enough for the complete conversion of **91a** with high selectivity toward the formation of **90**. Importantly, the reaction can proceed in water as the only solvent using H_2_O_2_ as oxidant and at moderate temperatures of 50–80 °C. In a follow-up study [104], they showed that different *N*-substituted glyphosates **91a**–**d** can undergo *N*-dealkylation using an Au/C catalyst; **91a** has the highest conversion and selectivity compared to the other starting compounds. More importantly, the byproduct of the *N*-dealkylation of **91a** is acetone which can be easily separated and recycled [104,107] (Figure 13).

It is noteworthy to mention in this section that besides the application of gold and platinum catalysts, a few other strategies for the *N*-dealkylation of **91** were also reported [108,109,110]. For example, Parry et al. [108] patented a procedure for the *N*-dealkylation of **91f** by acid hydrolysis using concentrated aqueous hydrobromic or hydroiodic acid (46–48% *w*/*w*) to remove the benzyl group preparing glyphosate with 41% yield. A similar approach was also patented [109] using strong acids (48% HCl, HI or HBr) for the *N*-dealkylation of **91d** with a yield of 95% of glyphosate. The Pt- and Au-catalyzed *N*-dealkylation methods were developed to avoid the application of concentrated acids in these processes. 

### 3.4. Ruthenium-, Rhodium- and Copper-Catalyzed

Oxidative *N*-dealkylation of amines is one of the important reactions specific to cytochrome P450 enzymes. Different studies have been performed for the simulation of this enzymatic activity, investigating different metal complexes of ruthenium, [111,112,113], rhodium [114,115] and copper [116,117,118,119]. 

**Figure 14:**
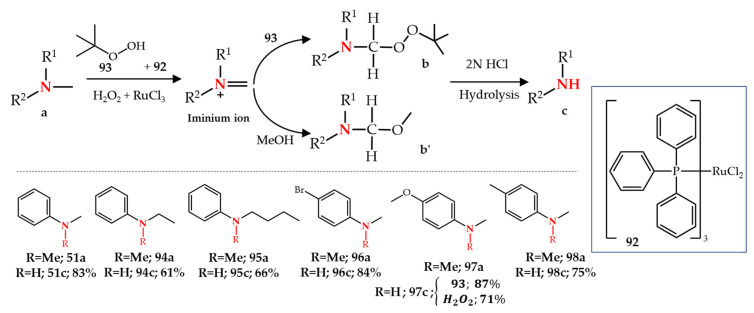
Ru-catalyzed *N*-dealkylation of amines.

Murahashi et al. [111] reported the first application of ruthenium for the *N*-dealkylation reaction and they recently reviewed the general applications of ruthenium-catalyzed oxidative transformations of different functionalities including the C-N bond in amines [120]. In this study, a ruthenium complex (RuCl_2_(PPh_3_)_3_ **92**) was used as catalyst for the oxidative *N*-demethylation of tertiary amines using *t*-BuOOH **93** as oxidant. Although generally the oxidation of tertiary amines with hydroperoxides in the presence of a transition-metal catalyst leads to *N*-oxide formation, they obtained the corresponding *α*-(*tert*-butyl-dioxy)alkylamines (Figure 14, intermediate **b**) as the product in the presence of a Ru catalyst and *t*-BuOOH. Subsequently, acid hydrolysis (HCl, 2 N) of these intermediates led to *N*-dealkylation and high yields of secondary amines **51c** and **94c**–**97c**. Following their quest for the simulation of the enzymatic function of amine monooxygenases with ruthenium-complex systems, Murahashi et al. [112] also reported another synthetic route for the *N*-demethylation of methylamines, using hydrogen peroxide as oxidant and RuCl_3_ as catalyst in methanol. Similar to their previous study, the *N*-demethylation is a two-step reaction in which the products of the Ru-catalyzed oxidation of amines **97a** and **98a** are their corresponding methoxymethylamine derivatives (Figure 14, compound **b′**) which upon the hydrolysis with 2 N HCl are converted to *N*-demethylated products **97c** and **98c**. The mechanistic studies suggested that the Ru(II) complex reacts with oxidants (*t*-BuOOH or H_2_O_2_) to give Ru(IV) = O. The oxidation of a tertiary amine by Ru(IV) = O leads to an iminium ion intermediate which either reacts with a second molecule of *t*-BuOOH as nucleophile and leads to intermediate **b** or reacts with MeOH to form intermediate **b′** (Figure 14). 

**Figure 15:**
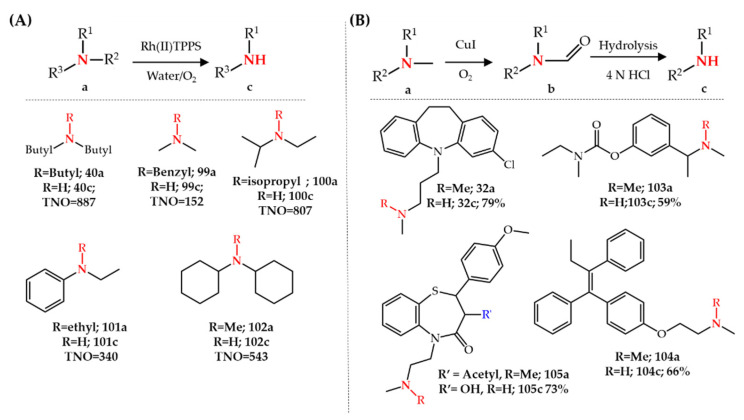
(**A**) Rhodium-catalyzed and (**B**) copper-catalyzed *N*-dealkylation of amines.

Fu et al. [114,115] reported the aerobic oxidative *N*-dealkylation of amines using a rhodium porphyrin (Rh(II)TPPS or Rh(II) of **79**) in aqueous solution at room temperature. Although various tertiary amines were examined in this study, the yield of the produced secondary amine was not reported. However, the turnover number of the reaction, TNO, which is the molar ratio of the product to the catalyst, was reported. Importantly, only 0.05% of this catalyst was used and a stochiometric amount of an acid such as HCl or CF_3_COOH (0.5–1 equivalent) was needed to perform the reaction. In contrast to the ruthenium-catalyzed system, rhodium-catalyzed *N*-dealkylation produces the *N*-dealkylated product in one step as well as allowing the removal of different alkyl groups such as methyl, ethyl, isopropyl, butyl and benzyl (compounds **40**, and **99**–**102**) (Figure 15A). 

Genovino et al. [118,119] reported a Cu-catalyzed oxidation of tertiary amines in pharmaceuticals to obtain *N*-dealkylated metabolites. Interestingly, the product of CuI/O_2_-catalyzed oxidation of a tertiary amine was a formamide structure which can be hydrolyzed under acidic conditions to the corresponding secondary amine. Among different investigated copper salts, CuI was found to be the most efficient catalyst for the oxidation of tertiary amines. A 20 mol% of catalyst at a temperature of 120 °C was enough for the synthesis of the formamide intermediate while concentrated HCl (4 N) at 100 °C was required for the hydrolysis step. A comparison between Cu(I)- and Cu(II)-catalyzed reactions in the absence of oxygen (under nitrogen atmosphere) revealed that the reaction proceeds in the presence of Cu(II) but not Cu(I). This observation supported the mechanistic hypothesis including the in situ formation of Cu(II) from CuI/O_2_ leading to a single electron oxidation of amines and the reduction of Cu(II) to Cu(I). *N*-demethylated metabolites of different pharmaceuticals such as clomipramine **32a**, rivastigmine **103a**, tamoxifen **104a**, and diltiazem **105a** were obtained using this method. Recently, Liu et al. [121] also reported the formation of formamide upon the oxidation of *N*,*N*-dimethylanilines using a CuI/O_2_ system (Figure 15B).

## 4. Electrochemical *N*-Dealkylation

Electroorganic synthesis uses electrons as an inexpensive, benign and renewable oxidant or reductant instead of chemical reagents, providing a highly sustainable alternative route for the synthesis of a broad range of chemicals based on green chemistry principles. Besides the green aspects of organic electrochemistry, it drastically reduces the number of chemical steps required by traditional reagents. Over the past two decades, organic electrochemistry has experienced a renaissance and has received growing interest. Therefore, the general application of organic electrochemistry including the transformation of the C-N bond has recently been reviewed in detail in numerous reports [5,122,123,124,125]. Moreover, electrochemical conversions provide an attractive approach for the in vitro simulation of drug metabolism and predicting the formation of potential metabolites, especially when electrochemical reactors are coupled with mass spectrometry systems [126,127]. 

**Figure 16:**
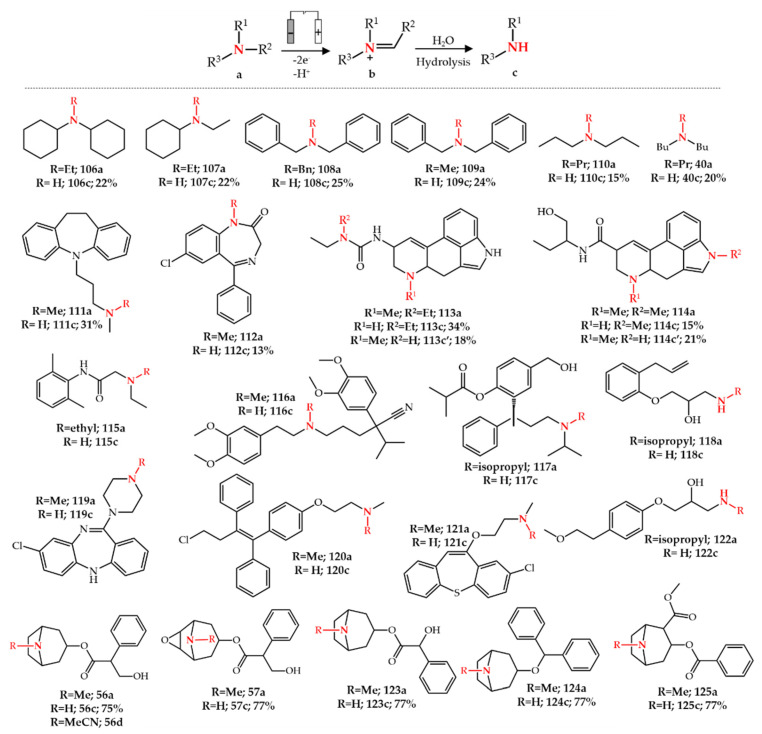
Electrochemical *N*-dealkylation of various pharmaceuticals.

Electrochemical oxidation of amines was studied as early as the 1960s by the anodic oxidation of triethylamine [128] and by recording cyclic voltammograms of some aliphatic amines [129,130] using platinum electrodes. Following these reports, Mann et al. [131,132,133] reported the electrochemical *N*-dealkylation of various tertiary amines (**40a**, **106a**–**110a**) in a three-electrode system using platinum and Ag/AgNO_3_ as working electrode and reference electrode, respectively. This study showed the potential of electrochemical *N*-dealkylation for the removal of methyl, ethyl, propyl, butyl, and benzyl groups. Shono et al. [134,135] later applied this method for the synthesis of *N*-dealkylated metabolites. A platinum working electrode and a saturated calomel reference electrode were used for the electrolysis while methanol was used as solvent in the presence of sodium hydroxide. *N*-dealkylated metabolites of various pharmaceuticals including imipramine **111a**, diazepam **112a**, lisuride **113a** and methysergide **114a** were obtained. Interestingly, **113a** and **114a** each generated two *N*-dealkylated metabolites upon the cleavage of R^1^ or R^2^ (Figure 16). It is hypothesized that the electrochemical oxidation of amines is triggered by a one electron transfer followed by a proton/electron transfer resulting in an iminium intermediate. Subsequent hydrolysis of the iminium intermediate leads to the *N*-dealkylated amine. This method was later applied to other nitrogen-containing chemical functionalities [125], and generally, the electrochemical oxidation route to activate or functionalize C-H bonds adjacent to a nitrogen atom through electron/proton/electron transfers is called Shono oxidation. Different studies were reported for electroanalytical *N*-dealkylations or the simulation of drug metabolism using the EC-MS (electrochemistry-mass spectrometry) approach [136,137,138,139,140,141,142,143,144,145]. These studies were carried out for analytical rather than synthetic purposes to obtain the metabolite profile of specific pharmaceuticals. For example, lidocaine **115a,** one of the most studied drugs using EC-MS, is converted to its *N*-dealkylated metabolite in the human body [138,139,140,141,146]. Gul et al. [141] showed that the electrochemical oxidation of **115a** at pH 12 using a glassy carbon working electrode leads to **115b** as the main product. Other studies also reported the electrochemical generation and subsequent MS analysis of *N*-dealkylated metabolites of other drugs such as verapamil **116a** [137], fesoterodine **117a** [142], alprenolol **118a** [147], clozapine **119a** [148], toremifene **120a** [149], zotepine **121a** [150], and metoprolol **122a** [151] (Figure 16).

**Figure 17:**
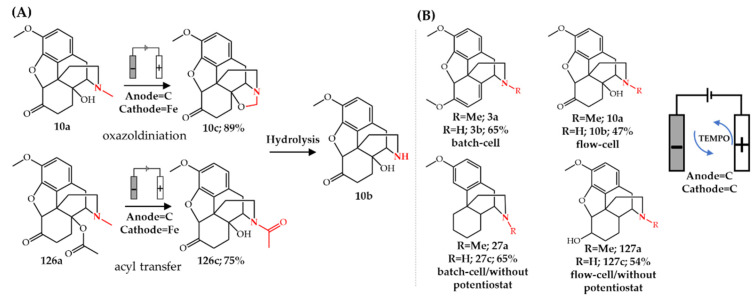
(**A**) Electrochemical oxazolidination and acyl transfer and (**B**) TEMPO-mediated electrochemical *N*-demethylation of opiate alkaloids.

Besides the reported drug metabolism studies, electrochemical *N*-dealkylation is also applied for the synthesis of pharmaceutical intermediates. We reported that the electrochemical *N*-demethylation of tropane alkaloids is a selective, facile and scalable approach for the synthesis of nortropane alkaloids (**56c**–**57c**, **123c**–**125c**) [152] (Figure 16). As discussed in Section 3.2, noratropine **56c** and noscopolamine **57c** are valuable intermediates for the semi-synthesis of **58** and **59**. A detailed description of how a two-electrode electrochemical cell can be fabricated using a low-cost porous glassy carbon material (100 pores per inch, PPI) was presented in this study. The reaction proceeds in one step, in 70% aq. ethanol at room temperature and can be conveniently scaled up to gram-scale synthesis of nortropane alkaloids. Due to the selectivity of the electrochemical reaction, the final reaction mixture is clean enough to avoid the need for chromatographic purification for isolation of the final product. A three-step liquid-liquid extraction was used to isolate **56c** in gram scale with 79% overall yield. Importantly, no *N*-demethylation was observed at neutral pH when using HCl salts of the tropane alkaloids. Therefore, tropane alkaloids were used in their free amine form to result in a high pH in the solution (pH = 10–12). As was also reported by Shono et al. [134,135] and Gul [141], the high pH facilitates electron abstraction from nitrogen during the electrochemical oxidation. Mechanistic studies supported the formation of an iminium intermediate. The electrochemical *N*-demethylation of **56a** in the presence of cyanide ions (by adding KCN) led to the formation of *N*-nitrilo noratropine **56d** by trapping the iminium intermediate [152]. Glotz et al. [153] showed that the electrochemical oxidation of opiates with a C14-hydroxyl group, such as **10a**, leads to an oxazolidine structure **10c** which was also observed for iron and palladium catalytic systems. Moreover, they showed that an electrochemical intramolecular acyl transfer for opiates with a C14-acyl group such as *O*-acetyloxycodone **126a** occurs similarly to palladium-catalyzed systems (see Section 3.1 and Section 3.2). Subsequent hydrolysis of **10c** or **126c** leads to noroxycodone **10b**. A two-electrode batch or flow electrochemical cell was used in this study as well as using graphite as anode and stainless steel as cathode [153] (Figure 17A).

We recently showed that a TEMPO-mediated electrochemical strategy affords the *N*-demethylation of opiate alkaloids in one step [12]. This reaction proceeds in 70% aq. acetonitrile at room temperature using a two-electrode reactor in which both electrodes were glassy carbon (100 PPI). Different noropiates with and without C14-hydroxyxl groups (**3b**, **10b**, **27c**, and **127c**) were electrochemically synthesized using TEMPO as electron mediator in both batch and flow conditions without the need for a supporting electrolyte in high yields. A low-cost DC-to-DC electrical converter connected to a solar-powered battery was used to replace the potentiostat by performing gram-scale synthesis of **27c** using this system. Divided-cell electrochemical experiments and subsequent LC-MS analysis of anodic and cathodic compartment solutions showed that the reaction only occurs in the anodic cell, supporting the formation of an iminium intermediate as mentioned earlier [12] (Figure 17B). Frazier et al. [154] patented an electrolytic process for glyphosate **90** production. They reported a brief description of electrochemical *N*-dealkylation of *N*-benzyl-*N*-phosphonomethyl glycine **91f** in flow conditions using porous graphite as anode, a carbon rod as cathode, and concentrated hydrochloric acid as solvent and supporting electrolyte, without reporting the conversion efficiency or yield [154] (Figure 13).

## 5. Photochemical *N*-Dealkylation

**Figure 18:**
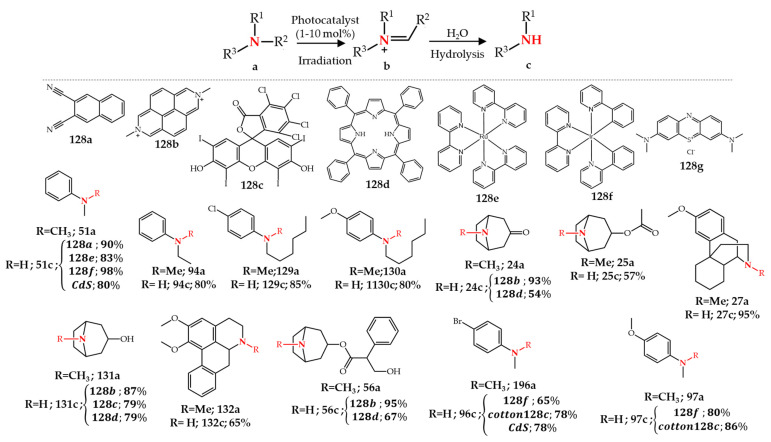
Photochemical *N*-dealkylation of various tertiary amines.

Different photochemical *N*-dealkylation methods using various photocatalysts have been developed for the synthesis of various *N*-dealkylated chemicals [155,156,157,158,159,160,161,162,163]. An early report by Pandey et al. [160] showed that the photolysis of a tertiary *N*-methyl amine using dicyanonaphtalene **128a** as electron acceptor in the presence of sodium hydroxide in methanol led to high yields of *N*-demethylated products (**51c**, **94c**, **129c**, and **130c**) (Figure 18). Santamaria et al. [161] showed that photochemical oxidation of different alkaloids such as **24a**, **27a 56a**, **131a**, and **132a** under oxygen atmosphere in the presence of *N*,*N*′-dimethyl-2,7-diazapyrenium difluoroborate **128b** as electron acceptor resulted in *N*-demethylated alkaloids with excellent yields. It was proposed in these early studies that an iminium intermediate is formed upon the photochemical oxidation of a tertiary amine, which is then hydrolyzed to a secondary amine (Figure 18). Ripper et al. [162] used the previous strategy for the synthesis of noropiates and nortropanes, but instead of **128b** they used Rose Bengal **128c** or TPP (meso-tetraphenylporphyrin) **128d** as photocatalysts. Although this procedure was successful for the *N*-demethylation of tropane alkaloids **24a**, **25a**, **56a**, and **131a** (Figure 18), photochemical *N*-demethylation of opiate alkaloids **27a** and **50a** did not lead to any *N*-demethylated products but resulted in various byproducts. Recently, Chen et al. [163] showed that photochemical oxidation of oxycodone resulted in the same oxazolidine structure that can be obtained by metal-catalyzed (Section 3) or electrochemical (Section 4) methods. In this method, **128c** was used as photocatalyst while bubbling oxygen through the reaction solution and using a LED source for irradiation. A 2-g scale synthesis of **10b** from **10a** upon the photochemical oxidation and subsequent hydrolysis by HCl (1 M, MeOH) in flow conditions led to 88% yield of noroxycodone **10b**.

Metal complexes such as the ruthenium or iridium complexes **128e** and **128f** have also been applied as photocatalysts for the *N*-demethylation of *N*,*N*-dimethylaniline derivatives **51a**, **96a**, and **97a**. In this method the presence of 1,4-diazabicyclo [2.2.2]octane (DABCO) as additive increased the yield of the final product while using only 1 mol% of **128e** or **128f** [164] (Figure 18). An innovative technique for recycling and reuse of photocatalyst **128c** was developed recently by immobilizing **128c** via an ionic bond onto cotton. Cotton fibers are an abundant natural material carrying hydroxyl groups on their surface which enables facile functionalization with **128c**. The cotton-**128c** photocatalyst was then applied for the *N*-demethylation of *N*,*N*-dimethylaniline derivatives such as **96a** and **97a** [165] (Figure 18). The presence of DABCO and acetic acid was necessary to perform *N*-demethylation reaction. This photocatalyst is recyclable and reusable by removing it from the reaction solution [165]. Recently, Firoozi and Sarvari [166] designed and synthesized a heterogenous and recyclable photocatalyst, cadmium sulfide (CdS) nanoparticles, for the photochemical *N*-demethylation of tertiary amines. This reaction also requires DABCO (10 mol%) as additive beside CdS (10 mol%). The reaction proceeds at room temperature under air (atmospheric pressure) using sunlight or blue LED irradiation. Besides *N*-demethylation, this method is capable of removing ethyl and butyl groups. Importantly, they showed the reusability of the CdS photocatalyst by five times recycling and reusing it for the synthesis of **51c** with negligible difference in isolated yield [166] (Figure 18).

**Figure 19:**
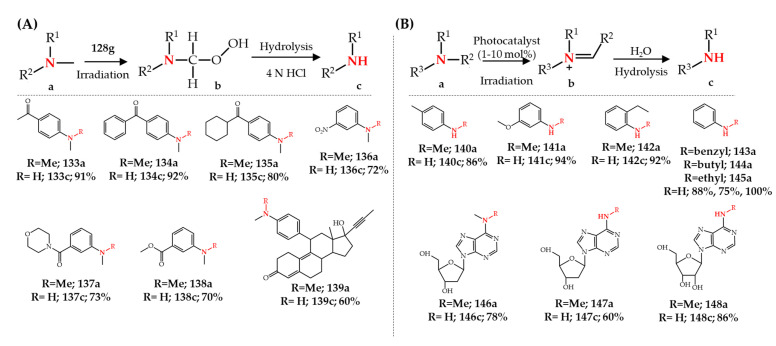
(**A**) Two-step photochemical *N*-demethylation of tertiary *N*-methylamines and (**B**) photochemical *N*-demethylation of secondary amines.

A two-step acetic acid promoted photochemical *N*-demethylation method using Rose Bengal **128c** or methylene blue **128g** as photocatalysts was developed for the *N*-demethylation of a wide range (up to 30 examples) of *N*,*N*-dimethylaminophenyl derivatives **133a**–**138a**. A hydroperoxide intermediate (Figure 19A, compound **b**) was formed upon the photochemical oxidation of tertiary amines which in a second step was hydrolyzed in acidic methanol (3 N H_2_SO_4_) to obtain the corresponding secondary amines. Beside the presence of DABCO, the addition of up to 25 equivalents of acetic acid increased the conversion of **133a** from 12% to 98%. This method was used in the gram-scale synthesis of the *N*-demethylated metabolite of mifepristone **139a** in 60% yield [167] (Figure 19A).

In addition to the previously discussed photochemical approaches for the *N*-dealkylation of tertiary *N*-alkyl-anilines, Zhao and Leonori [168] recently showed that this approach can also be applied for the *N*-dealkylation of secondary *N*-alkyl-anilines. Iridium complex **128f** was applied as the photocatalyst while a blue LED was used as the light source for irradiation. Importantly, an amine such as triethylamine or piperidine was added to the reaction solution without which no *N*-dealkylation was observed for secondary amines. It was hypothesized that the presence of triethylamine or piperidine led to the formation of a hydrogen bond between the nitrogen of the additive and the -NH of aniline increasing the electron density on the nitrogen atom of the secondary aniline. Therefore, it was expected that this phenomenon decreases the oxidation potential of the secondary aniline, which indeed was observed by cyclic voltammetry experiments. A very broad range of secondary amines (46 examples), such as **140a**–**145a**, were successfully *N*-dealkylated to obtain primary amines by removing various alkyl groups such as methyl, ethyl, butyl, and benzyl [168]. An interesting application of photochemistry was also reported recently for the photochemical *N*-demethylation of *N*^6^-methyl groups in *N*^6^-methyl adenines **146a**–**148a** (Figure 19B). *N*^6^-methyl adenosine **148** is the most abundant internal modification in eukaryotic mRNA and some RNA demethylases such as the AlkHB5 proteins repair this modification in vivo. In this study, riboflavin (vitamin B_2_) was used as a photocatalyst under LED light irradiation [169].

## 6. Enzymatic *N*-Dealkylation

The identification and structural elucidation of various metabolites of a newly discovered drug candidate is one of the most important steps during drug discovery and development studies. Different in vivo and in vitro methods such as human and animal microsomes, animal models, or isolated enzymes are used for the investigation of metabolic pathways of a drug candidate [170]. Enzymes of the Cytochrome P450 (CYP) superfamily are important enzymes for the metabolism of xenobiotics, as they are involved in more than 75% of all drug metabolism. These heme-containing enzymes catalyze various metabolic transformations comprising the *N*-dealkylation reaction [127]. The application of CYP enzymes for analytical drug metabolism studies have been reported a number of times and have been reviewed in detail [171,172,173,174,175,176]. Besides the analytical study of drug metabolism, the synthesis of drug metabolites in preparative scale is also important for metabolite activity and toxicity studies. However, this is impractical due to the limited availability of CYP enzymes in models such as liver microsomes. To overcome this problem, different approaches for the expression of CYP enzymes in other living organisms, such as bacteria, have been developed and were recently reviewed as well [177,178,179,180]. Therefore, recent research reporting biocatalytic *N*-dealkylation for the analysis or synthesis of *N*-dealkylated compounds will be briefly presented here.

**Figure 20:**
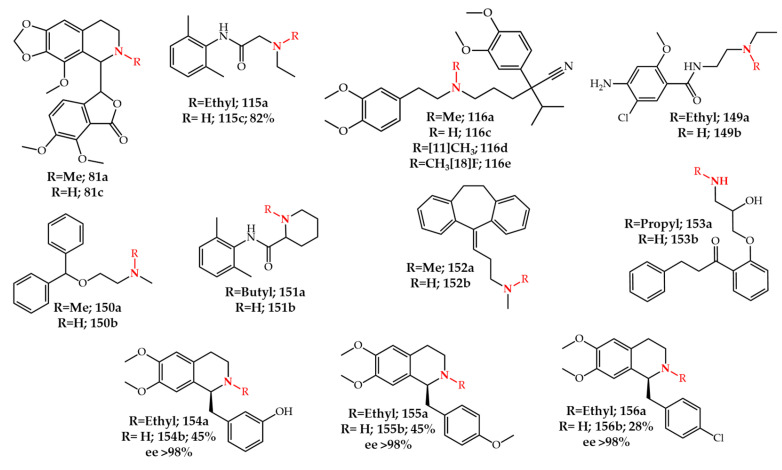
Examples of substrates undergoing enzymatic *N*-dealkylation.

Verapamil **116a**, a calcium channel blocker which is mainly used for cardiovascular disorders, is transformed to its *N*-demethylated metabolite in vivo. Human and rat liver microsome studies showed that norverapamil **116c** is the major product [181]. Besides its importance as a metabolite, **116c** also acts as a reversing agent of multi-drug resistance in chemotherapy by inhibition of P-glycoproteins. Therefore, its synthesis in preparative scale is of importance. A recent report by Shen et al. [182] has identified a new CYP enzyme from a bacterium (*Streptomyces griseus* ATCC 13273) which efficiently carries out *N*-demethylation of **116a** to **116c**. Moreover, **116c** can be used as a precursor for the synthesis of ^13^C-verapamil **116d** or ^18^F-verapamil **116e** which are used as positron emission tomography (PET) tracers for the investigation of P-glycoprotein function in the blood-brain barrier [183,184]. *N*-dealkylated metabolites of other drugs such as metoclopramide **149a** [185], diphenhydramine **150a** [186], bupivacaine **151a** [187], amitriptyline **152a** [188], and propafenone **153a** [189] were also identified using human or animal liver microsomes, or isolated CYP enzymes (Figure 20).

Besides analytical studies, some reports have used CYP enzymes for synthetic applications [190,191,192,193]. Ren et al. [190] developed a CYP mutant library based on P450_BM3_ (CYP102A1) from *Bacillus megaterium* which enables the *N*-dealkylation reaction. Using some variants from this library, lidocaine **115a** and amitriptyline **152a** were converted to **115c** and **152b** with 82% and 96% yield, respectively. Richards et al. [192,193] also used a mutant library of P450_BM3_ for the synthesis of *N*-demethylated noscapine **81c**. Upon the screening of different CYP mutants, a specific mutant showed 88% selectivity toward the *N*-demethylation reaction. Subsequently, they incorporated the mutant enzyme into a whole-cell biotransformation process by employing *Bacillus megaterium* to reach 27.5 mg/L as the highest productivity for the synthesis of **81c**.

Other biocatalytic approaches besides those using CYP enzymes have also been recently applied for the *N*-dealkylation reaction [194,195,196,197]. For example, Gandomkar et al. [194] reported an enantioselective oxidative aerobic *N*-dealkylation using berberine bridge enzyme. When racemic mixtures of **154a**–**156a** were used as starting reactants, only (*S*)-**154b**–**156b** were obtained as products with an optical purity (ee) of more than 98%. Augustin et al. [197] identified a microorganism capable of opiate *N*-demethylation transformation (Thebainfresser, a Methylobacterium) by culturing the sludge waste obtained from an opium poppy processing facility in Tasmania. Their thorough investigation led to the discovery of MND (morphinan *N*-demethylase) which retained its activity in different organic solvents while *N*-demethylating a broad scope of compounds such as opiate and tropane alkaloids.

## 7. Conclusions

In this review, we have surveyed the literature to provide an overview of methods for the *N*-dealkylation of amines. This reaction is of utmost importance for the synthesis of different pharmaceuticals and agrochemicals on an industrial scale. Moreover, the *N*-dealkylation reaction is important for identification and synthesis of drug metabolite as those are required throughout all phases of drug development studies and their synthesis is still a challenge. Besides the traditional chemical methods, various methods are reported for this reaction, applying transition-metal catalysts, electrochemistry, photochemistry and enzymes.

## Data Availability

Not applicable.
